# Supplementary material for: High-resolution structures with bound Mn2+ and Cd2+ map the metal import pathway in an Nramp transporter
Source: eLife. 2023 Apr 11;12:e84006. doi: 10.7554/eLife.84006 (PMC10185341; doi:10.7554/eLife.84006)
Supplement: Supplementary file 1. — (a) Construct, precipitant, and soaking solutions used for each structure. (b) Data collection and refinement statistics for the four supporting new DraNramp structures. (c) Cα RMSD in Å for all DraNramp structure pairs (number of aligned residues in parentheses). (d) Data collection statistics for anomalous maps. (e) Distances to metal (Å) for coordinating atoms at the orthosteric site. (f) Coordination number and geometry of metal ions in the orthosteric site. (g) Binding affinity of metals to various DraNramp constructs. (h) Primers for Mutagenesis (5’ to 3’ sequence). (i) Summary of molecular dynamics simulations. [file elife-84006-supp1.docx]

**High-resolution structures with bound Mn^2+^ and Cd^2+^ map the metal import pathway in an Nramp transporter**

**Shamayeeta Ray^1^, Samuel P. Berry^1^, Eric A. Wilson^2^, Casey H. Zhang^1,3^, Mrinal Shekhar^4^, Abhishek Singharoy^2^, and Rachelle Gaudet^1^***

**Affiliations**

^1^Department of Molecular and Cellular Biology, Harvard University, Cambridge, MA USA

^2^School of Molecular Sciences, Arizona State University, Tempe, AZ USA

^3^Present address: Grossman School of Medicine, New York University, New York, NY USA

^4^Broad Institute of MIT and Harvard, Cambridge, MA USA

* To whom correspondence should be addressed (gaudet@mcb.harvard.edu)

**This file contains Supplementary Tables 1-9**

**Supplementary file 1a.** **Construct, precipitant, and soaking solutions used for each structure**

| Structure (Construct•substrate)^a^ | Precipitant solution | Soaking solution^c^ |
| --- | --- | --- |
| ΔN31-WT (no metal) | 28% PEG 400  0.1 M MES (pH 6.7)  50 mM succinic acid (pH 6.0)  5 mM spermidine (pH 7.0) |  |
| ΔN31-WT_soak_ | 28% PEG 400  0.1 M MES (pH 6.3)  50 mM succinic acid (pH 6.0)  5 mM spermidine (pH 7.0) | 28% PEG 400  0.1 M MES (pH 6.5)  50 mM succinic acid (pH 6.0)  20 mM spermidine (pH 7.0) |
| ΔN31­-WT•Mn^2+^ | 24% PEG 400  0.1 M MES (pH 5.9)  50 mM succinic acid (pH 6.0)  20 mM spermidine (pH 7.0) | 28% PEG 400  0.1 M MES (pH 6.5)  50 mM succinic acid (pH 6.0)  20 mM spermidine (pH 7.0)  2 mM MnCl_2_ |
| ΔN31-WT•Cd^2+^ | 24% PEG 400  0.1 M MES (pH 6.1)  50 mM succinic acid (pH 6.0)  20 mM spermidine (pH 7.0) | 28% PEG 400  0.1 M MES (pH 6.5)  50 mM succinic acid (pH 6.0)  20 mM spermidine (pH 7.0)  2 mM CdCl_2_ |
| A47W•Mn^2+ (b)^ | 20% PEG MME 550  0.1 M HEPES (pH 7.2)  0.25 M NaCl  10 mM MnCl_2_ |  |
| ΔN31-M230A•Mn^2+ (b)^ | 32% PEG 400  0.1 M MES (pH 6.5)  50 mM succinic acid (pH 6.0)  20 mM spermidine (pH 7.0)  10 mM MnCl_2_ |  |
| ΔN31-D296A•Mn^2+ (b)^ | 28% PEG 400  0.1 M HEPES (pH 6.8)  0.1 M NaCl  10 mM MnCl_2_ |  |

^a^ ΔN31 refers to a deletion of the N-terminal 31 residues. For simplicity, the “ΔN31” annotation is listed here, but omitted elsewhere in the text.

^b^ Proteins were premixed with 5 mM MnCl_2_ prior to setting up crystallization trials in precipitants spiked with 10 mM MnCl_2_.

^c^ Crystals of ‘ΔN31-WT (no metal)’ were soaked in the listed soaking solution overnight prior to harvesting.

**Supplementary file 1b.** **Data collection and refinement statistics for the four supporting new DraNramp structures**

| Structure | WT | A47W•Mn^2+^ | D296A•Mn^2+^ | G223W•Mn^2+ (a)^ |
| --- | --- | --- | --- | --- |
| Conformation | Occluded | Occluded | Inward open | Outward open |
| Bound substrate | none | Mn^2+^ | Mn^2+^ | Mn^2+^ |
| PDB ID | 8E5S | 8E6H | 8E6L | 8E6N |
| **Data Collection** |  |  |  |  |
| Beamline | NECAT 24IDC | NECAT 24IDC | GMCA 23IDB | NECAT 24IDC |
| Wavelength (Å) | 0.984 | 0.984 | 1.033 | 0.979 |
| Resolution range (Å) | 41.06-2.38 (2.46-2.38) | 45.35-2.39 (2.47-2.39) | 45.32-3.12 (3.23-3.12) | 39.19-2.40 (2.49-2.40) |
| Space group | P 2 21 21 | P 2 21 21 | P 2 21 21 | C1 21 |
| Unit cell (*a, b, c*) | 58.66, 70.85, 98.34 | 58.98, 70.93, 98.57 | 58.51, 71.64, 98.89 | 105.76, 80.39, 51.75 |
| Unit cell (α, β, γ) | 90, 90, 90 | 90, 90, 90 | 90, 90, 90 | 90, 94.72, 90 |
| Number of crystals | 1 | 1 | 3 | ~15 |
| Total reflections | 74541 (7226) | 61843 (6302) | 27350 (2837) | 49321 |
| Unique reflections | 15762 (1607) | 16817 (1653) | 7004 (687) | 13962 (621) |
| Redundancy | 4.7 (4.5) | 3.7 (3.8) | 3.9 (4.1) | 3.5 (1.7) |
| Completeness (%) | 91.66 (93.38) | 98.57 (99.52) | 89.46 (90.75) | 82.1* (36.4) |
| Mean *I/σ (I)* | 9.83 (1.60) | 7.92 (1.03) | 6.71 (1.49) | 6.2 (1.6) |
| Wilson *B*-factor | 36.73 | 40.33 | 63.48 | 58.94 |
| *R*_merge_ | 0.192 (1.057) | 0.163 (1.371) | 0.342 (1.216) | 0.186 |
| *R*_meas_ | 0.218 (1.189) | 0.191 (1.588) | 0.386 (1.380) | 0.21 |
| *R*_pim_ | 0.099 (0.528) | 0.097 (0.789) | 0.173 (0.634) | 0.095 |
| CC1/2 | 0.99 (0.51) | 0.98 (0.41) | 0.80 (0.53) | 0.98 (0.58) |
| **Refinement** |  |  |  |  |
| Resolution range (Å) | 41.06-2.38 (2.46-2.38) | 45.35-2.39 (2.47-2.39) | 45.32-3.12 (3.23-3.12) | 39.19-2.40 (2.49-2.40) |
| No. reflections | 15665 (1579) | 16749 (1653) | 6999 (687) | 13963 (621) |
| No. reflections in *R*_free_ | 1565 (159) | 1673 (165) | 910 (90) | 701 (33) |
| *R*_work_ | 0.206 (0.264) | 0.205 (0.299) | 0.219 (0.287) | 0.223 (0.276) |
| *R*_free_ | 0.248 (0.312) | 0.246 (0.371) | 0.272 (0.352) | 0.271 (0.337) |
| Number of atoms | 3403 | 3483 | 3205 | 3310 |
| Protein | 2922 | 2939 | 2861 | 3012 |
| Ligand | 434 | 469 | 323 | 244 |
| Water | 47 | 75 | 21 | 54 |
| Protein Residues | 392 | 392 | 385 | 398 |
| Ramachandran plot |  |  |  |  |
| Favored (%) | 98.72 | 99.74 | 98.16 | 97.73 |
| Allowed (%) | 1.28 | 0.26 | 1.84 | 2.27 |
| Outliers (%) | 0 | 0 | 0 | 0 |
| Rotamer outliers (%) | 0.34 | 1.00 | 0.69 | 1.29 |
| Clashscore | 7.21 | 6.38 | 7.04 | 6.48 |
| RMS (bonds) | 0.003 | 0.002 | 0.002 | 0.002 |
| RMS (angles) | 0.49 | 0.43 | 0.45 | 0.44 |
| Average *B*-factor | 48.09 | 50.38 | 68.03 | 75.00 |
| Protein | 46.20 | 47.30 | 68.42 | 74.11 |
| Ligand | 61.54 | 70.13 | 65.22 | 88.03 |
| Water | 41.47 | 47.56 | 58.65 | 65.83 |
| No. of TLS groups | 7 | 3 | 8 | 5 |

Values in parentheses are for highest-resolution shell. Data for D296A•Mn^2+^ merge reflections from multiple crystals. Data for the other structures were obtained from a single crystal.

^a^ G223W•Mn^2+^ has been re-refined from PDB ID: 6BU5, the previously published data collection statistics (Bozzi, Zimanyi, et al., 2019) are reproduced here for completeness.

**Supplementary file 1c. Cα RMSD in Å for all DraNramp structure pairs (number of aligned residues in parentheses)**

| **Conformation** |  | **Outward open** | | **Occluded** | | | | | **Inward open** | | |
| --- | --- | --- | --- | --- | --- | --- | --- | --- | --- | --- | --- |
|  | **Structure** | G223W ^(a)^ | G223W•Mn^2+^ | WT_soak_ | WT | WT•Mn^2+^ | A47W•Mn^2+^ | Patch ^(a,b)^ | M230A•Mn^2+^ | D296A•Mn^2+ (b)^ | WT•Cd^2+^ |
| **Outward open** | G223W ^(a)^ |  | 0.97 (396) | 2.29  (359) | 2.29  (356) | 2.33  (361) | 2.39  (359) | 2.57  (320) | 2.45  (356) | 2.38  (344) | 2.32  (347) |
|  | G223W•Mn^2+^ | 0.97  (396) |  | 2.34  (348) | 2.38  (350) | 2.39  (351) | 2.47  (353) | 2.67  (320) | 2.62  (355) | 2.47  (339) | 2.47  (398) |
| **Occluded** | WT_soak_ | 2.29  (359) | 2.34  (348) |  | 0.38  (391) | 0.20  (392) | 0.43  (388) | 1.54  (328) | 0.56  (386) | 0.61  (366) | 0.77  (372) |
|  | WT | 2.29  (356) | 2.38  (350) | 0.38  (391) |  | 0.39  (392) | 0.42  (391) | 1.69  (329) | 0.71  (387) | 0.82  (367) | 0.95  (374) |
|  | WT•Mn^2+^ | 2.33  (361) | 2.39  (351) | 0.20  (392) | 0.39  (392) |  | 0.47  (389) | 1.53  (328) | 0.59  (387) | 0.65  (367) | 0.79  (373) |
|  | A47W•Mn^2+^ | 2.39  (359) | 2.47  (353) | 0.43  (388) | 0.42  (391) | 0.47  (389) |  | 1.59  (326) | 0.59  (383) | 0.61  (363) | 0.60  (368) |
| **Inward open** | Patch ^(a,b,c)^ | 2.57  (320) | 2.67  (320) | 1.54  (328) | 1.69  (329) | 1.53  (328) | 1.59  (326) |  | 1.59  (339) | 1.45  (340) | 1.52  (341) |
|  | M230A•Mn^2+^ | 2.45  (356) | 2.62  (355) | 0.56  (386) | 0.71  (387) | 0.59  (387) | 0.59  (383) | 1.59  (339) |  | 0.38  (372) | 0.48  (376) |
|  | D296A•Mn^2+ (b,c)^ | 2.38  (344) | 2.47  (339) | 0.61  (366) | 0.82  (367) | 0.65  (367) | 0.61  (363) | 1.45  (340) | 0.38  (372) |  | 0.47  (384) |
|  | WT•Cd^2+ (c)^ | 2.32  (347) | 2.47  (398) | 0.77  (372) | 0.95  (374) | 0.79  (373) | 0.60  (368) | 1.52  (341) | 0.48  (376) | 0.47  (384) |  |
| ^a^ Previously published structures (G223W is PDB ID: 6D91; Patch is PDB ID: 6D9W and so named because it has a patch of mutations in intracellular loops)  ^b^ Lower-resolution structures.  ^c^ Structures with unmodeled loops. | | | | | | | | | | | |

**Supplementary file 1d. Data collection for anomalous maps**

|  | A47W•Mn^2+^ | D296A•Mn^2+^ | WT•Cd^2+^ |
| --- | --- | --- | --- |
|  | Occluded | Inward open | Inward open |
| Beamline | NECAT 24IDC | GMCA 23IDB | NECAT 24IDC |
| Wavelength (Å) | 0.984 | 1.033 | 1.904 |
| Resolution range (Å) | 45.35-2.38 (2.46-2.38) | 45.32-3.12 (3.23- 3.12) | 40.28-2.82 (2.92-2.82) |
| Space group | P 2 21 21 | P 2 21 21 | P 2 21 21 |
| Unit cell (*a, b, c)* | 58.98, 70.93, 98.57 | 58.51, 71.64, 98.89 | 58.54, 70.72, 98.00 |
| Unit cell (α, β, γ) | 90, 90, 90 | 90, 90, 90 | 90, 90, 90 |
| Total reflections | 62633 (6419) | 27350 (2837) | 32558 (3197) |
| Unique reflections | 17027 (1685) | 7000 (688) | 10017 (989) |
| Redundancy | 3.7 (3.8) | 3.9 (4.1) | 3.3 (3.2) |
| Completeness (%) | 98.61 (99.53) | 89.48 (90.89) | 96.78 (97.63) |
| Mean *I/σ (I)* | 7.83 (1.00) | 6.71 (1.49) | 6.96 (1.27) |
| Wilson *B*-factor | 40.44 | 63.59 | 54.2 |
| *R*_merge_ | 0.166 (1.409) | 0.321 (1.216) | 0.165 (1.024) |
| *R*_meas_ | 0.191 (1.633) | 0.398 (1.380) | 0.195 (1.219) |
| *R*_pim_ | 0.099 (0.813) | 0.232 (0.634) | 0.101 (0.643) |
| CC1/2 | 0.98 (0.38) | 0.80 (0.53) | 0.98 (0.35) |
| Anomalous completeness (%) | 89.9 (90.6) | 78.1 (77.9) | 85.8 (84.8) |
| Anomalous multiplicity (%) | 1.8 (2.0) | 1.9 (2.3) | 1.6 (1.8) |

Values in parentheses are for highest-resolution shell. Mn^2+^ ions were co-crystallized with the protein, whereas Cd^2+^ ions were soaked after crystallization (Supplementary Table 1).

**Supplementary file 1e. Distances to metal (Å) for coordinating atoms at the orthosteric site**

|  | **Outward open** | **Occluded** | | **Inward open** | | |  |
| --- | --- | --- | --- | --- | --- | --- | --- |
| **Coordinating atom** | G223W•Mn^2+^ | WT•Mn^2+^ | A47W•Mn^2+^ | M230A•Mn^2+^ | D296A•Mn^2+ e^ | WT•Cd^2+^ | |
| D56 Oδ1 | 2.4 | 2.7 | 2.6 | 2.7 | 2.7 |  | |
| N59 Oδ1 | 3.2 | 2.4 | 2.5 | 2.6 | 2.4 | 2.9 | |
| M230 Sδ | 3.0 | 2.8 | 2.8 |  | 2.7 | 3.4 | |
| A227 O (Cα) |  | 2.5 | 2.2 | 2.4 | 2.2 | 2.8 | |
| A53 O (Cα) | 2.4 | 2.2 | 2.1 |  |  |  | |
| Y54 O (Cα) |  |  |  | 3.1 | 3.5 | 3.2 | |
| water O (Q378)^a^ | 2.7 | 2.3 | 2.2 | 2.5 | 2.3 | 2.9 | |
| water O (M230A)^b^ |  |  |  | 2.3 |  |  | |
| water (Y54)^c^ |  |  |  | 2.2 |  | 3.3 | |
| water (N59)^d^ | 2.6 |  |  |  |  |  | |
| ^a^ Conserved water coordinating central binding ion (water/metal) and Oε1 of Q378 (except in G223W•Mn^2+^ where Q378 is far away to be in coordinating distance).  ^b^ Water replacing M230 in M230A•Mn^2+^ structure.  ^c^ Water coordinating central metal ion and Cα carbonyl of Y54.  ^d^ Water coordinating central metal ion and Oδ1 and Nδ2 of N59 in G223W•Mn^2+^.  ^e^ Missing coordinating bonds due to low resolution of the D296A•Mn^2+^ structure. | | | | | | | |

**Supplementary file 1f.** **Coordination number and geometry of metal ions in the orthosteric site**

|  | | **Coordination number** | **Coordinating atoms** | **Geometry** | **RMS_angle_ (°)** |
| --- | --- | --- | --- | --- | --- |
| **Outward open** | G223W•Mn^2+^ | 6 | SO_5_ | Octahedral | 22 |
| **Occluded** | WT•Mn^2+^ | 6 | SO_5_ | Octahedral | 25 |
|  | A47W•Mn^2+^ | 6 | SO_5_ | Octahedral | 23 |
| **Inward open** | M230A•Mn^2+^ | 7 | O_7_ | Pentagonal bipyramidal | 36 |
|  | D296A•Mn^2+^* | 6 (7) | SO_5_ | Pentagonal bipyramidal | 36 |
|  | WT•Cd^2+^ | 6 | SO_5_ | Octahedral | 34 |
| *Missing postulated coordinating bond to water molecule due to the low resolution of the D296A•Mn^2+^ structure. | | | | | |

**Supplementary file 1g.** **Binding affinity of metals to various DraNramp constructs**

| **Protein construct** | **Manganese** | | | **Cadmium** | | |
| --- | --- | --- | --- | --- | --- | --- |
|  | **No of sites** | **K_d_ (µM)** | | **No of sites** | **K_d_ (µM)** | |
| WT | 2 | 190 ± 30 | 1970 ± 520 | 2 | 55 ± 15 | 220 ± 20 |
| A47W | 2 | 125 ± 5 | 2450 ± 650 | 1 | 150 ± 10 | |
| D56A | 2 | 230 ± 80 | 3800 ± 1100 | 2 | 80 ± 20 | 2250 ± 750 |
| M230A | 2 | 215 ± 65 | 4600 ± 1300 | 1 | 160 ± 20 | |
| G223W | 1 | 440 ± 15 | | No binding | | |
| D296A | 1 | 370 ± 30 | | 1 | 120 ± 1 | |
| D369A | 1 | 420 ± 30 | | 1 | 70 ± 10 | |
| A47W-D296A | 1 | 255 ± 45 | | No binding | | |
| A47W-D369A | 1 | 300 ± 95 | | No binding | | |
| D56A-D296A | 1 | 250 ± 80 | | No binding | | |
| D56A-D369A | 1 | 210 ± 70 | | No binding | | |
| M230A-D296A | 1 | 230 ± 90 | | No binding | | |
| M230A-D369A | 1 | 770 ± 20 | | No binding | | |

The number of sites were assigned as described in and Appendix 1. Each K_d_ value represents the mean of 2-3 independent ITC experiments and the mean ± SEM is reported (see Appendix 1 for the results of all individual experiments).

**Supplementary file 1h. Primers for Mutagenesis (5’ to 3’ sequence)**

| **Mutation** | **Primer** | **Sequence** |
| --- | --- | --- |
| A47W | forward | GGCCGTGGGTCATCGCGTCTATCGCCTACATG |
|  | reverse | TGACCCACGGCCCGAGAAACGGCAGGATGC |
| D296A | forward | GGCGCCCTGACCACCGCCTAC­CAGAC |
|  | reverse | GGTCAGGGCGCCCGCGTTTTCCACGTTCTT |
| D369A | forward | ATGGCCCCGTCGTCGGTGCTGATCTTGTCG |
|  | reverse | CGACGGGGCCATGCCCAGCAGAATGACGAT |
| Y54A | forward | CGTCTATCGCCGCCATGGACCCCGGCAAC |
|  | reverse | CCATGCCGGCGATAGACGCGATGACCGCC |
| Y54F | forward | CGCCTTCATGGACCCCGGCAACTTTGCG |
|  | reverse | CCATGAAGGCGATAGACGCGATGACCGCC |
| M230A | forward | CGGTCGCCCCACACGTCATCTACCTGCACTCGGC |
|  | reverse | TGTGGGGCGACCGTCGCCCCGATGATGCCCAC |
| Q89A | forward | GCGATGGTGATTGCGAACCTCAGCGCC |
|  | reverse | GGCGCTGAGGTTCGCAATCACCATCGC |
| H232A | forward | ATGCCAGCCGTCATCTACCTGCACTCGGCGCTC |
|  | reverse | GACGGCTGGCATGACCGTCGCCCCGATGAT |
| H237A | forward | TACCTGGCCTCGGCGCTCACGCAGGGACGC |
|  | reverse | CGAGGCCAGGTAGATGACGTGTGGCATGACCGT |

**Supplementary file 1i.** **Summary of molecular dynamics simulations**

| **Simulation** | **Time (ns)** | **Starting structure** | **Number of atoms** |
| --- | --- | --- | --- |
| S1a | 1031.95 | Outward-open, G223W•Mn^2+^ (PDB ID 6BU5) with Mn^2+^ removed and residue 223 mutated back to native glycine *in silico* | 104,624 |
| S1b | 617.55 |  |  |
| S2a | 769.80 | New WT•Mn^2+^ inward-occluded structure, with Mn^2+^ removed | 103,074 |
| S2b | 1022.85 |  |  |
| S3a | 1176.00 | New WT•Cd^2+^ inward-open structure, with Cd^2+^ removed | 103,372 |
| S3b | 808.80 |  |  |
